# Exploring the association between vitamin C intake and gingival bleeding tendency in healthy, non‑deficient young adults

**DOI:** 10.1002/jper.70142

**Published:** 2026-06-04

**Authors:** Thijs M. H. de Jong, Dagmar E. Slot, Cees Valkenburg, Bruno G. Loos, Fridus A. van der Weijden

**Affiliations:** ^1^ Department of Periodontology Academic Centre for Dentistry Amsterdam (ACTA) University of Amsterdam and Vrije Universiteit Amsterdam Amsterdam The Netherlands

**Keywords:** bleeding, gingival bleeding, gingivitis, nutrition, vitamin C

## Abstract

**Background:**

The aim of this study is to investigate the relationship between reported vitamin C intake and gingival bleeding severity.

**Methods:**

In this cross‐sectional study, 336 participants were screened, and 262 (118 females, 144 males) were eligible. Vitamin C intake was derived from the Food Frequency Questionnaire (FFQ). The body mass index (BMI), plaque index (PI), bleeding on marginal probing (BOMP), and bleeding on pocket probing (BOPP) were scored. Means and standard deviations (SD) were calculated overall and by sex. Spearman's correlations assessed links between vitamin C intake and clinical variables. Sex differences were analyzed using Fisher's exact test and the Mann‐Whitney *U* test. Generalized regression models evaluated relationships between BOMP/BOPP and vitamin C intake, sex, age, and PI.

**Results:**

The participants had a mean age of 22.6 years, mean BMI of 22.9 kg/m^2^, and mean PI of 0.98. Average daily vitamin C intake was 108 mg (SD = 53), ranging from 19 to 425 mg. Mean BOMP and BOPP scores were 0.38 and 0.52, respectively. Regression models revealed no significant associations between vitamin C intake and BOMP or BOPP. However, PI was positively associated with both bleeding indices, while age was negatively associated.

**Conclusion:**

In this non‑deficient young adult population, vitamin C intake was not significantly associated with gingival bleeding after adjusting for covariates. Gingival bleeding was positively associated with PI and negatively with age.

## Plain language summary

This study looked at whether the amount of vitamin C that people consume is linked to how much their gums bleed, a common sign of gum inflammation. We examined more than 260 young adults, asking them about their diets and checking their oral health, including how much plaque they had and how much their gums bled during dental exams. While people's vitamin C intake varied widely, we found no clear connection between how much vitamin C they consumed and how much their gums bled. However, we did find that people with more plaque on their teeth tended to have more gum bleeding, and younger participants had slightly more bleeding than older ones. These findings suggest that, at least in this group of healthy young adults, vitamin C intake does not play a major role in gum bleeding. Instead, keeping plaque under control seems to be more important for gum health.

## 1 INTRODUCTION

Ascorbic acid, originally known as hexuronic acid, is an organic compound with the formula C_6_H_8_O_6_, commonly referred to as vitamin C. It serves as an indispensable nutrient for humans, who are incapable of endogenous synthesis. Unlike many other species, humans lack the functional enzyme L‐gulonolactone oxidase, which is necessary for the synthesis of vitamin C, necessitating its acquisition through dietary sources.^1^ Vitamin C functions as a potent antioxidant, playing an essential role in reducing oxidative stress.^2^ It is integral to various physiological processes, particularly collagen synthesis, where it serves as a specific electron donor for enzymes involved in collagen hydroxylation.^3^ This inability to synthesize ascorbic acid forms the basis for deficiency‐related conditions, as severe vitamin C deficiency is associated with scurvy related periodontitis^4^ and necrotizing periodontitis.^5^ Furthermore, inadequate plasma levels of vitamin C are associated with increased inflammation, suggesting a need for closer examination of those at risk for inflammation‐driven diseases.^6^

Periodontal disease encompasses various inflammatory oral pathologies, with the most prevalent being gingivitis and periodontitis. Gingivitis is an inflammatory process of the gingiva that is reversible and serves as a precursor to periodontitis.^7^, ^8^, ^9^ Therefore, gingivitis is considered a risk factor for periodontal attachment loss and tooth loss.^7^ Gingival tissues with gingivitis rarely show spontaneous bleeding but can be provoked to bleed by probing the gingival margin with a blunt instrument.^7^

It was demonstrated that, in a population of young, non‐smoking adults, daily consumption of either 200 g of guava or 200 mg of synthetic vitamin C, administered both prior to and during a period of oral hygiene abstention, had a preventive effect on the development of experimental gingivitis. In contrast, the control group exhibited the typical progression of gingival inflammation. However, other studies have not consistently supported a clear association between vitamin C intake and gingival bleeding.^10^, ^11^, ^12^

Hence, the interest in examining the relationship between vitamin C intake and gingivitis remains. In this study, a broad cross‐sectional assessment was conducted among 262 non‐smoking, systemically healthy young adults within a relatively narrow age range. In this study the focus was to evaluate the correlation between vitamin C intake and the extent of gingival bleeding as clinically assessed following probing.

## 2 MATERIALS AND METHODS

### 2.1 Study design and ethical procedures

The current study is a secondary analysis of a project that was designed as a cross‐sectional, single‐center, observational clinical study. It was conducted at the Academic Centre for Dentistry Amsterdam, The Netherlands, as part of the project “Estimating the Boundaries for a Healthy Oral Ecosystem in Young Individuals,” within the framework of the Top Institute Food and Nutrition.^13^, ^14^, ^15^, ^16^, ^17^, ^18^ This study adhered to the principles outlined in the Declaration of Helsinki (2008) by the World Medical Association and Good Clinical Practice guidelines. The medical ethics committee of the Academic Medical Centre of Amsterdam approved the protocol (2012_210#B2012406), and it was registered in the Dutch Trial Register (NTR3649). Informed consent forms were signed by all participants.

### 2.2 Study population

This study examined a convenience sample comprising healthy non‐dental students from universities in and around Amsterdam who had voluntarily registered in a database expressing interest in participating in clinical dental‐oriented research. Recruitment took place through emails and flyers, with screening of potential volunteers commencing from October 2012 to March 2013. Participants were selected based on a completed medical questionnaire to establish their systemic health status. Selection criteria included having visited a general dentist within the past year and being considered free of oral or dental issues.

Further screening procedures involved oral examinations and scoring of the Dutch Periodontal Screening Index (DPSI)^19^, ^20^ by dental care professionals. The inclusion criterion for participants was a DPSI score of minus ≤ 3, which implies a maximum probing depth of 5 mm in the absence of gingival recession. Gingival bleeding and the presence of calculus were not taken into account. The enlistment protocol, as well as the inclusion and exclusion criteria, have been extensively outlined in prior studies.^13^, ^14^ Specifically, the exclusion criteria encompassed: smoking (>1 cigarette a day for the last year), evident dental caries, oral infections, removable partial denture or night guard, (peri)oral piercings, drug abuse, self‐reported pregnancy or breastfeeding, the presence of systemic disease, recent use of antibiotics in the past 3 months, and the use of anti‐inflammatory drugs or other prescribed medications (except for oral contraceptives) that might interfere with the outcomes of this study.

### 2.3 Procedures

Participants were instructed to abstain from consuming food, beverages, gum chewing, or engaging in strenuous physical exercise prior to their appointments. Additionally, they were instructed to avoid oral hygiene procedures for 24 hours leading up to the appointment and to cease eating, including chewing gum and drinking, after midnight the day before. The assessments for each individual were conducted on a single day and followed a predetermined order.

### 2.4 Vitamin C intake

To evaluate macro and micronutrient intake, frequency of food consumption, the use of sodas and juices, a validated general food frequency questionnaire (FFQ) was administered.^21^, ^22^, ^23^, ^24^ The FFQ covers a period of 1 month (4 weeks) and was developed based on the most recent Dutch National Food Consumption Survey and the Dutch food composition table (NEVO).^25^ It includes questions on the frequency and amount of intake in household measures of 75 food items commonly consumed in the Dutch diet. In addition to food items, the FFQ included questions on the use of nutritional supplements during the preceding month, including vitamin C. Total vitamin C intake was calculated by combining vitamin C derived from foods and supplements. The conversion from food consumption to energy and nutrient intake is established with a computerized version of the Dutch Food Composition table NEVO and includes, among other nutrients, vitamin C. The quantification of each food item was based on an approximate weight, and the portion size was determined per gram of the respective food item. The total daily intake of vitamin C was then calculated without distinguishing between dietary and supplemental sources.

### 2.5 Physical parameters

From each participant, the weight in kilograms was determined using an electronic digital scale, rounded to one decimal point. Height measurements were taken with the participant in a straight standing position without shoes, against a wall, recorded in centimeters. Subsequently, the Body Mass Index (BMI) was calculated by dividing body weight (in kilograms) by the square of height (in meters).^26^

### 2.6 Oral parameters

The clinical measurements were carried out by trained examiners, each responsible for a single index. Bleeding on marginal probing (BOMP) and dental plaque were assessed in two randomly selected contralateral quadrants (one upper and one lower).^27^ Each tooth was examined at six sites per tooth (disto‐vestibular, vestibular, mesio‐vestibular, distolingual, lingual, and mesio‐lingual, excluding third molars). The randomization for quadrant selection was performed using true random numbers generated by atmospheric noise (www.random.org). For bleeding on pocket probing (BOPP), all four quadrants were examined. The order of measurements was as follows:

1. BOMP: scored in two quadrants (by Examiner 1). The gingivae were lightly dried with compressed air, and a ball‐ended probe (Ash Probe EN15, Dentsply International, New York, USA) was inserted into the gingival crevice to a depth of approximately 2 mm or until slight resistance was felt. The probe was then gently run along the gingival margin at an angle of approximately 60° and in contact with the oral sulcular epithelium until the next interproximal area, at the vestibular and lingual surfaces, respectively. The number of gingival units that bled upon probing were recorded using scores from 0 to 2 (0 = non‐bleeding, 1 = pin prick, 2 = excessive) within 30 s after probing.^28^, ^29^
2. BOPP: full mouth examination in four quadrants (by Examiner 2). A 0.25N force‐controlled probe (Hawe Neos Click‐Probe, KerrHawe, Bioggio, Switzerland) was inserted parallel to the root surface directed apically toward the perceived location of the root apex at each of the six sites. The number of pocket units that bled upon probing were recorded (0 = non‐bleeding, 1 = bleeding) within 30 s after probing.
3. Dental plaque: scored in two quadrants (by Examiner 3) according to the Silness & Loe plaque index (PI) (0–3).^30^, ^31^

### 2.7 Statistical analysis

Data were analyzed using R statistical software (version 4.2.2; R Foundation for Statistical Computing, Vienna, Austria). A significance level of ≤0.05 was used for all statistical tests. Spearman's rank correlation coefficients were computed to assess the relationship between vitamin C intake and the clinical parameters of gingival bleeding, BOMP and BOPP, and the variables age, BMI, and PI. These analyses were stratified by sex to explore potential differences in correlation patterns between males and females. Interpretation of the Spearman's rank correlation coefficients followed Cohen's guidelines, as described in detail by Rowntree.^32^

To examine sex differences across the variables, Fisher's Exact Test was used for categorical variables to ensure robustness. The Mann‐Whitney *U* test, appropriate for continuous or ordinal variables that do not follow a normal distribution, was used to compare sex with daily vitamin C intake, BOMP, BOPP, age, BMI, and PI.

Generalized linear models (GLMs) were applied to further explore the associations between the clinical parameters of gingival bleeding (BOMP or BOPP) and vitamin C intake, sex, age, and PI. A gamma distribution with a logarithmic link function was chosen when the outcome variables were non‐normally distributed. To facilitate comparability of effect sizes, continuous predictors (vitamin C intake, age, and PI) were standardized by centering them at their mean and scaling them by their standard deviation (i.e., *z*‐scoring). This transformation ensures that regression coefficients represent the effect of a one standard deviation increase in the predictor variable on the outcome. The regression coefficients, standard errors, *t*‐values, and *p*‐values were reported for each predictor. Interaction terms were included to explore potential moderating effects of sex. Model fit was assessed using Akaike's Information Criterion (AIC) and residual diagnostics. Additionally, analysis of variance (ANOVA) was conducted to evaluate the significance of interaction effects.

To provide context for interpreting the findings, a sensitivity power analysis was performed for BOMP and BOPP. Correlation coefficients (*r*) were interpreted according to Rowntree.^32^

## 3 RESULTS

### 3.1 Participant flow

**FIGURE 1:**
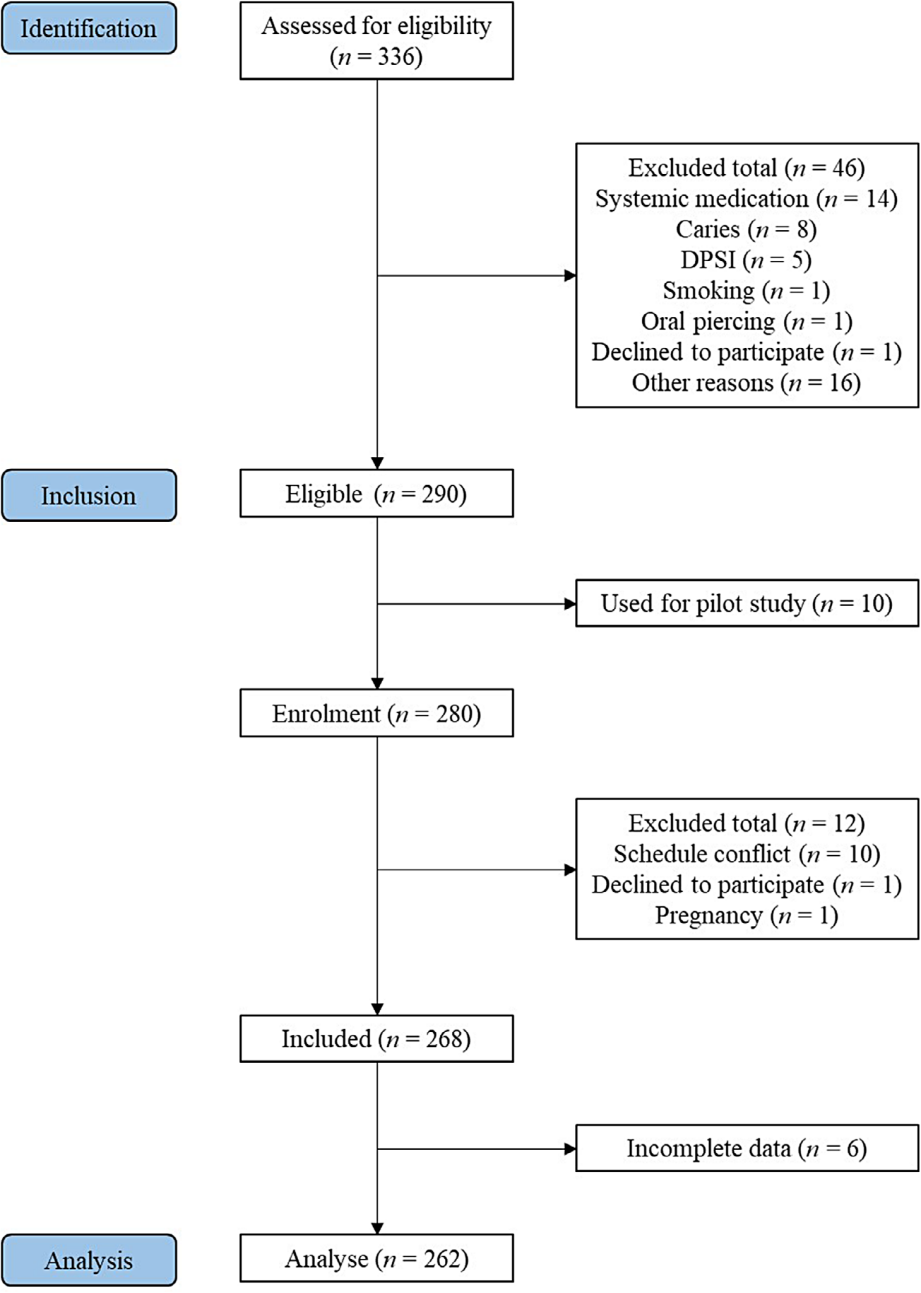
Flow chart of the inclusion process of study participants.

Figure 1 presents a flowchart of the initial 336 individuals assessed for eligibility. Based on the screening process, 46 participants were excluded. The first 10 subjects were used as a pilot to review and optimize the study workflow, evaluate the timing of assessments, and refine the standard operating procedures (SOPs). Data from these participants were excluded from further analysis to ensure consistency in the final dataset. From the remaining group, 12 subjects were excluded, primarily because they were unable to attend the scheduled appointment, and data from six individuals was incomplete. Consequently, 262 systemically healthy young adults were included based on a complete dataset.

### 3.2 Participant demographics

As seen in Table 1, the mean age of the included participants was 22.6 years, with a range from 18 to 32 years. The participants included 118 females (45%) and 144 males (55%). The mean BMI was 22.9 kg/m^2^ with a range from 16.7 to 35.7 kg/m^2^.

**TABLE 1:** Descriptive analysis of age, BMI, vitamin C intake, and various clinical parameters, overall and by sex.

	Overall (*N* = 262)		Female (*N* = 118)		Male (*N* = 144)		Statistics between sex
Variable	Mean (SD)	Range	Mean (SD)	Range	Mean (SD)	Range	*p*‐value
Age	22.6 (2.6)	18–32	22.6 (2.6)	18–31	22.5 (2.7)	18–32	0.484^a^
BMI (kg/m^2^)	22.9 (2.8)	16.7–35.7	22.6 (3.0)	16.7–35.7	23.2 (2.6)	17.7–34.2	**0.007** ^a^
Vitamin C (mg)	109 (53)	19–425	95 (40)	27–304	120 (60)	19–425	**<0.001** ^a^
BOMP	0.38 (0.25)	0.01–1.24	0.35 (0.21)	0.01–1.00	0.41 (0.26)	0.01–1.24	0.085^a^
BOPP	0.52 (0.15)	0.12–0.92	0.48 (0.15)	0.12–0.86	0.54 (0.15)	0.22–0.92	**0.003** ^b^
PI	0.98 (0.41)	0.02–2.10	0.87 (0.40)	0.02–2.10	1.07 (0.39)	0.15–2.07	**<0.001** ^b^

*Note*: Statistical significance was defined as *p* < 0.05.

Abbreviations: BMI, body mass index; BOMP, bleeding on marginal probing; BOPP, bleeding on pocket probing; kg, kilogram; m, meter; mg, milligram; PI, plaque index; SD, standard deviation.

^a^ Mann‐Whitney U‐test used.

^b^ Independent‐samples *t*‐test was used.

### 3.3 Vitamin C intake

**FIGURE 2:**
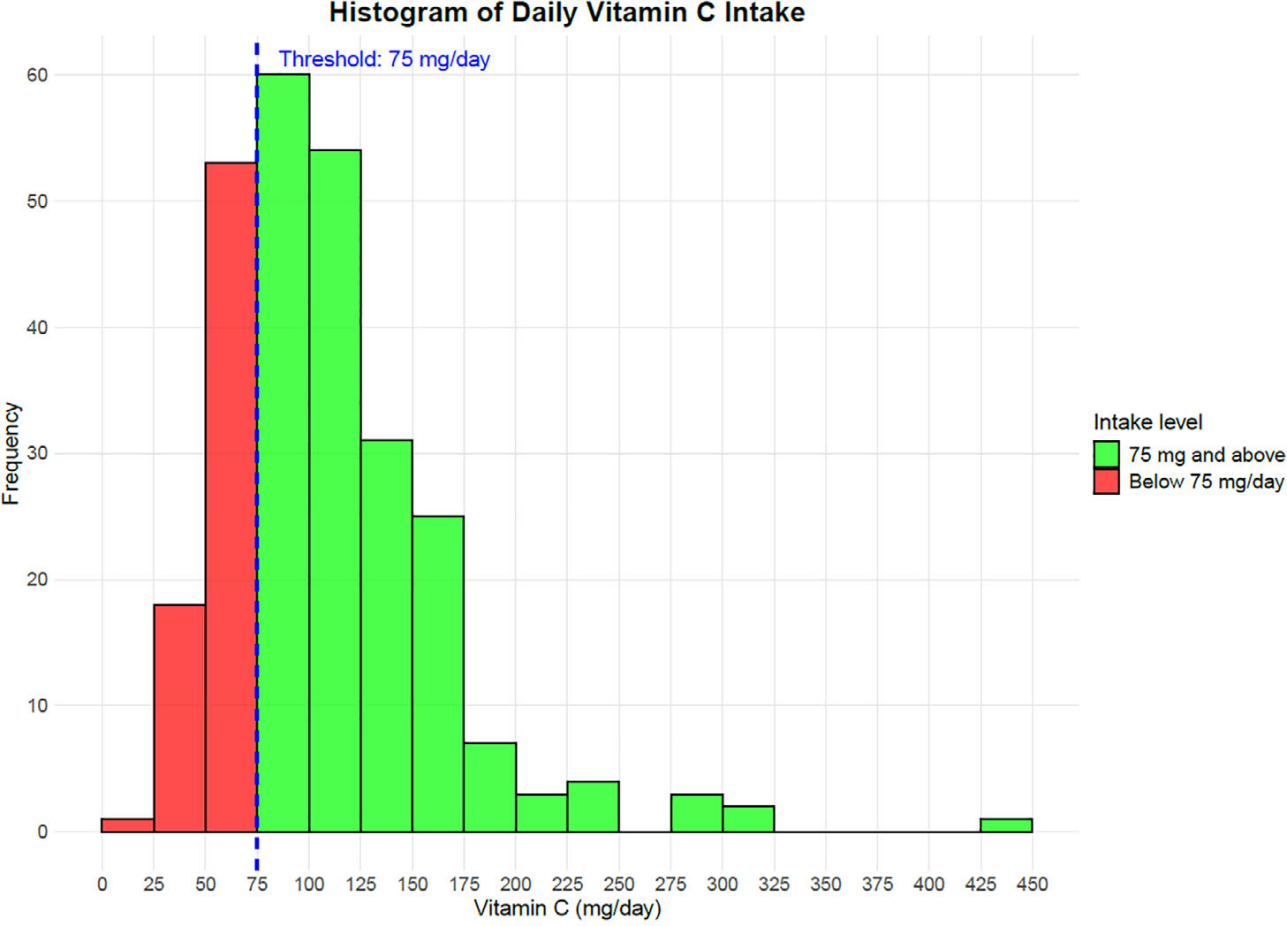
Histogram of reported daily vitamin C intake among participants. The x‐axis represents daily vitamin C intake in milligrams (mg), grouped into 25 mg intervals. The y‐axis indicates the number of participants within each intake range. A total of 190 out of 262 participants (73%) met or exceeded the recommended daily intake of 75 mg of vitamin C.

The mean daily vitamin C intake among participants was 108 mg, with individual intakes ranging from 19 to 425 mg (Table 1, Figure 2). Among female participants, the average intake was 95 mg per day (range: 26–303 mg), while male participants had a higher average intake of 119.92 mg per day (range: 19–425 mg). Overall, 190 of 262 participants (73%) met or exceeded the recommended daily intake of 75 mg of vitamin C and no participants had very low vitamin C intake (<10 mg/day) (Table S1 in the online *Journal of Periodontology*).

### 3.4 Gingival bleeding

**FIGURE 3:**
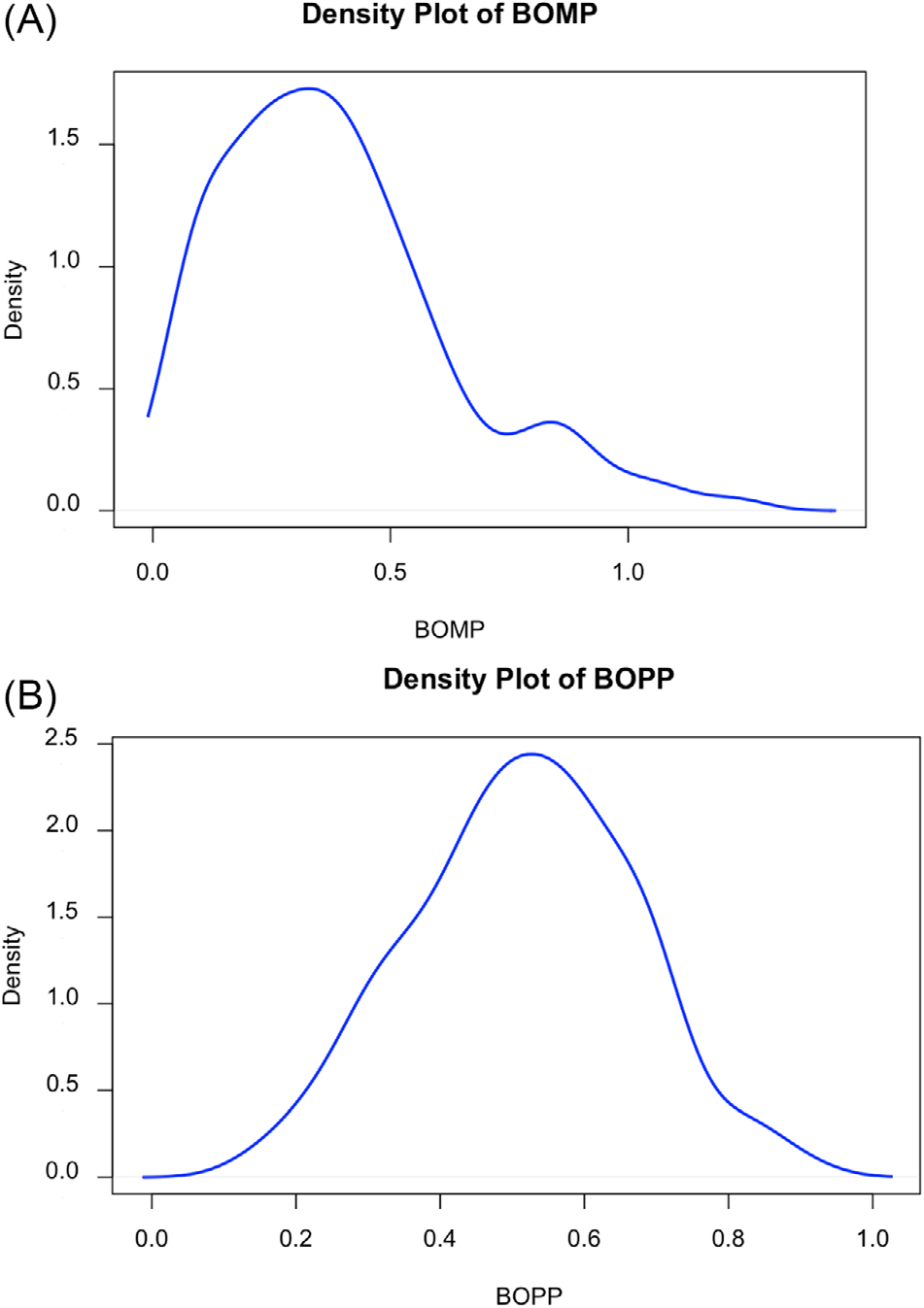
(A) Density plot of BOMP. The x‐axis represents BOMP scores, ranging from 0 to 2. The y‐axis shows density, which reflects the relative concentration of observations at each BOMP score, estimated using kernel density estimation. The area under the curve sums to 1, providing a normalized view of the distribution. The distribution of the BOMP was slightly skewed to the right. BOMP, bleeding on marginal probing. (B) Density plot of BOPP. The x‐axis represents BOPP scores, ranging from 0 to 1. The y‐axis shows density, which reflects the relative concentration of observations at each BOPP score, estimated using kernel density estimation. The area under the curve sums to 1, providing a normalized view of the distribution. The distribution of the BOPP was normally distributed. BOPP, bleeding on pocket probing.

The overall average BOMP was 0.38 with a range from 0.01 to 1.24 and the overall average BOPP was 0.52 with a range from 0.12 to 0.92 (Table 1). The distribution of the BOMP was slightly skewed to the right (Figure 3A), the BOPP was normally distributed (Figure 3B). Thirty participants had a BOMP score <  0.1, while none of the participants had a BOPP score <  0.1 (Table S1).

Table 1 shows that for females the average BOMP was 0.35 with a range from 0.01 to 1.00 and the average BOPP 0.48 with a range from 0.12 to 0.86. For males, the average BOMP was 0.41 with a range from 0.01 to 1.24 and the average BOPP 0.54 with a range from 0.22 to 0.92.

To provide a more clinically interpretable perspective on gingival inflammation, participants were categorized based on their mean BOMP and BOPP scores into approximate “healthy,” “localized,” and “generalized” bleeding groups (Table S2 in the online *Journal of Periodontology*). For BOMP, 11.5% of participants were classified as healthy (<0.1), 30.2% as localized (0.1–0.3), and 58.4% as generalized (≥0.3), whereas BOPP scores indicated no participants in the healthy range, 8.8% as localized, and 91.2% as generalized. These categorizations provide an approximate representation of the distribution of gingival bleeding within the cohort. However, formal gingivitis severity or a definitive periodontal diagnosis cannot be inferred from these indices alone, as clinical attachment level measurements were not available. Overall, this approach allows the presentation of gingival inflammation in a more clinically meaningful framework while remaining consistent with the limitations inherent to bleeding indices.

### 3.5 Plaque

The mean PI was 0.98 with a range from 0.02 to 2.10 (Table 1).

### 3.6 Sex differences

The BMI, vitamin C intake, BOPP, and the PI were significantly different between females and males (Table 1). Females had a lower BMI compared with males (on average 22.6 vs. 23.2 kg/m^2^), lower vitamin C intake (on average 95 vs. 120 mg), less BOPP (0.48 vs. 0.54), and a lower PI (0.87 vs. 1.07).

### 3.7 Correlation between vitamin C intake and gingival bleeding

Spearman's correlation analysis revealed only a significant but very weak correlation between vitamin C intake in the overall population and the clinical parameter of gingival bleeding BOPP. No statistically significant correlations were seen between vitamin C intake and the clinical parameter of gingival bleeding BOMP. Also, no statistically significant correlations were seen between vitamin C intake and age, BMI and PI, for the overall population, neither for females nor for males (Table 2).

**TABLE 2:** Correlation of vitamin C intake with age, BMI, and with various clinical parameters.

	Overall (*N* = 262)			Female (*N* = 118)			Male (*N* = 144)		
Variable	Spearman's correlation (rho)	*p*‐value	Strength of correlation^a^	Spearman's correlation (rho)	*p*‐value	Strength of correlation^a^	Spearman's correlation (rho)	*p*‐value	Strength of correlation^a^
Age	−0.075	0.226	NA	−0.116	0.210	NA	−0.038	0.648	NA
BMI (kg/m^2^)	0.033	0.595	NA	0.049	0.600	NA	0.009	0.913	NA
BOMP	0.005	0.937	NA	−0.036	0.696	NA	−0.017	0.842	NA
BOPP	0.127	**0.039**	Very weak	0.034	0.717	NA	0.109	0.192	NA
PI	0.050	0.418	NA	−0.027	0.770	NA	0.013	0.879	NA

*Note*: Statistical significance was defined as *p* < 0.05.

Abbreviations: BMI, body mass index; BOMP, bleeding on marginal probing; BOPP, bleeding on pocket probing; kg, kilogram; m, meter; NA, not applicable; PI, plaque index.

^a^ Spearman's rank correlation coefficients were interpreted according to Cohen's guidelines, as described in detail by Rowntree^32^; analyzing relationships: 0.0–0.2, very weak, negligible; 0.2–0.4, weak; 0.4–0.7, moderate; 0.7–0.9, strong, high, marked; 0.9–1.0, very strong, very high; NA if there is no significant relation.

### 3.8 Generalized regression models

Additionally, generalized regression models were used to examine the relationship between BOMP and BOPP with vitamin C intake adjusting for covariates, including sex, age, and PI. The results are presented in Tables 3 and 4. For vitamin C intake no significant association was observed with BOMP (estimate = 0.035, *p* = 0.372) and with BOPP (estimate = 0.007, *p* = 0.688). Also, sex (male vs. female) shows no association with BOMP (estimate = 0.115, *p* = 0.155) or with BOPP (estimate = 0.072, *p* = 0.054).

**TABLE 3:** Generalized regression model of BOMP and vitamin C intake, sex, age, and PI.

Parameter	Estimate	95% CI	Standard error	*t*‐value	*p*‐value
(Intercept)	−1.048	−1.163; –0.933	0.059	−17.902	**<0.001**
Vitamin C intake^a^	−0.035	−0.112; 0.042	0.039	−0.894	0.372
Sex (male vs. female)^b^	0.115	−0.043; 0.274	0.081	1.427	0.155
Age^a^	−0.090	−0.165; –0.015	0.038	−2.347	**0.020**
PI^a^	0.194	0.1171; 0.272	0.039	4.931	**<0.001**

*Note*: Statistical significance was defined as *p* < 0.05.

Abbreviations: CI, confidence interval; PI, plaque index; vs., versus.

^a^ Vitamin C intake, age, and PI were standardized (mean = 0, SD = 1) before analysis.

^b^ Sex is male, with female being the reference category in the regression analysis.

**TABLE 4:** Generalized regression model of BOPP and vitamin C intake, sex, age, and PI.

Parameter	Estimate	95% CI	Standard error	*t*‐value	*p*‐value
(Intercept)	−0.709	−0.762; –0.656	0.027	−26.357	**<0.001**
Vitamin C intake^a^	0.007	−0.028; 0.043	0.018	0.403	0.688
Sex (male vs. female)^b^	0.072	−0.001; 0.145	0.037	1.938	0.054
Age^a^	−0.050	−0.085; –0.016	0.018	−2.866	**0.004**
PI^a^	0.081	0.045; 0.117	0.018	4.467	**<0.001**

*Note*: Statistical significance was defined as *p* < 0.05.

Abbreviations: CI, confidence interval; PI, plaque index; vs., versus.

^a^ Vitamin C intake, age, and PI were standardized (mean = 0, SD = 1) before analysis.

^b^ Sex is male, with female being the reference category in the regression analysis.

The regression model for BOMP indicates that age (scaled) has a negative association with BOMP (estimate = –0.090, *p* = 0.020), and PI (scaled) has a positive association with BOMP (estimate = 0.194, *p* < 0.001). Similar results were seen for the regression model for BOPP that indicates that age (scaled) is negatively associated with BOPP (estimate = –0.050, *p* = 0.004), and PI (scaled) has a positive association with BOPP (estimate = 0.081, *p* < 0.001). In the regression model, a one‐standard deviation higher plaque index was associated with an approximately 20% higher level in BOMP and an 8% higher level in BOPP, indicating a modest but clinically meaningful magnitude of association despite the lack of a significant rank correlation.

### 3.9 Sensitivity analysis

Because this was a secondary analysis, the sample size (*n* = 262) was determined by the parent study. To aid interpretation of the findings, a sensitivity power analysis was performed for both bleeding outcomes (BOMP and BOPP), which were analyzed using comparable multivariable regression models with identical sample size and covariate structure. With a two‐sided *α* = 0.05, the study had 80% power to detect a small association corresponding to an absolute partial correlation of approximately *r* ≈ 0.17, equivalent to a partial *R* ^2^ of about 0.03 for vitamin C intake after adjustment for covariates. In practical terms, vitamin C would need to explain roughly 3% additional variance in the bleeding outcomes to be reliably detected.

The standardized regression coefficients observed for vitamin C in both models (*β* ≈ −0.03 to −0.07 for BOMP and *β* ≈ 0.002 for BOPP) were substantially smaller than this minimum detectable effect. Therefore, although no statistically significant associations were observed, very small effects of vitamin C on bleeding outcomes cannot be excluded.

## 4 DISCUSSION

### 4.1 Objective and findings

This study aimed to explore the relationship between vitamin C intake and gingival bleeding tendency. Spearman's correlation analysis revealed a significant, but very weak correlation between vitamin C intake and the clinical parameter of gingival bleeding BOPP and no significant correlations between vitamin C intake and BOMP. This suggests that within this population, vitamin C intake alone is not a strong predictor of gingival bleeding tendency. Significant differences in BMI and PI between sexes were observed, and males had a higher average daily vitamin C intake than females.

Generalized regression modeling of BOMP and BOPP adjusting for relevant covariates showed no significant association with vitamin C intake. The models indicated that plaque was positively associated with both BOMP and BOPP, while age was negatively associated with these clinical parameters.

### 4.2 Gingivitis

Gingivitis, characterized by a reversible inflammation of the gingiva without permanent damage to the supporting structures of the teeth, is considered a risk factor for periodontitis, the severe form of gum disease affecting the connective tissues and bone supporting the teeth.^7^ While gingivitis represents a normal and protective immune response by the host, periodontitis reflects a dysregulated or abnormal immune reaction.^33^ Although not all cases of gingivitis progress to periodontitis, the presence of gingivitis is a necessary precursor. Therefore, effectively managing gingivitis is an important preventive measure in reducing the risk of developing periodontitis.^34^

### 4.3 Vitamin C

Vitamin C is considered an essential nutrient requiring dietary intake, as humans lack the capacity to synthesize it.^1^ Based on population studies, plasma vitamin C concentrations have been categorized according to internationally recognized thresholds as follows: deficient (<11.3 µmol/L [<2.0 mg/L]), depleted (11.4–22.6 µmol/L [2.0–3.9 mg/L]), adequate/normal (22.7–56.7 µmol/L [4.0–9.9 mg/L]), and saturated (≥56.8 µmol/L [≥10.0 mg/L]).^35^ Acute vitamin C deficiency can lead to scurvy and clinical signs may begin to appear within 1 month of consuming less than 10 mg of vitamin C per day.^36^, ^37^ Vitamin C deficiency impairs collagen synthesis by disrupting the hydroxylation of proline and this may increase the permeability of the oral mucosa and gingival tissues to endotoxins.^3^, ^38^ Furthermore, it is demonstrated that vitamin C enhances the motility of polymorphonuclear leukocytes and may contribute to reducing tissue damage and inflammatory pathologies linked to NET formation,^39^ while its deficiency compromises host immune responses.^40^, ^41^, ^42^ Functioning as a potent antioxidant, vitamin C plays a crucial role in reducing oxidative stress.^2^ These findings suggest that vitamin C may influence periodontal health by supporting collagen turnover, maintaining epithelial barrier integrity, and modulating immune cell function.

### 4.4 Clinical parameters

In this study, both the BOMP and BOPP indices were employed to assess gingival bleeding tendency. The BOMP index, originally introduced by Löe,^43^ Ainamo and Bay,^44^ and Saxton and van der Ouderaa,^28^ involves gentle probing at the entrance of the gingival crevice, making it particularly suitable for assessing gingivitis. In contrast, BOPP is considered more reflective of the inflammatory processes associated with periodontitis.^45^ Previous research has demonstrated that probing to the base of the periodontal pocket results in significantly more bleeding than probing along the gingival margin. For the assessment of gingival health, BOMP is preferred, as it more accurately reflects inflammation associated with gingivitis.^13^, ^46^, ^47^ The BOMP index scores range from 0 to 2, while the BOPP ranges from 0 to 1. In our study the distribution of BOMP scores was more strongly skewed towards gingival health than that of BOPP. This outcome was expected, given the young and systemically healthy nature of the study population. The mean BOPP score was 0.52 (range: 0.12–0.92), whereas the mean BOMP score was lower at 0.38 (range: 0.01–1.24). These findings are consistent with those of Van der Weijden et al.,^46^ who reported similarly low BOMP scores (0.12–0.14) in a population of healthy dental students. In contrast, studies involving non‐dental students reported higher average BOMP scores, ranging from 0.80 to 1.56.^47^, ^48^

Interestingly, BOPP scores exhibited a more normal distribution, which does not fully align with the preselection criteria and the anticipated skew toward health. These findings suggest that BOMP may be the more sensitive index for detecting early gingival inflammation in populations with predominantly healthy periodontal status, whereas BOPP may overestimate the level of inflammation. The inclusion of participants with probing depths up to 5 mm could have introduced individuals with suboptimal periodontal health, contributing to variability in bleeding outcomes. Nonetheless, the relatively limited range of scores as assessed with both indices may reduce the sensitivity for detecting correlations, which may partially explain the absence of statistically significant associations between vitamin C intake and gingival bleeding in our analysis.

The higher prevalence of bleeding sites observed with BOPP is likely attributable, at least in part, to mechanical trauma rather than true inflammatory activity, particularly in this cohort of young adults with probing depths not exceeding 5 mm. As Lang et al.^49^ noted, trauma to clinically healthy gingival tissues is a common occurrence and may contribute to false‐positive bleeding responses. Moreover, the angulation of the probe plays a critical role in bleeding detection. Probing at an angle of approximately 60° to the long axis of the tooth has been shown to be more sensitive and specific for detecting gingival inflammation, while minimizing trauma‐induced bleeding, compared with vertical probing to the pocket base.^29^, ^46^

Several methodological factors may partly explain why BOPP scores appeared higher than BOMP scores, though the overall patterns observed remain informative. BOMP was recorded in two quadrants, whereas BOPP was assessed full‐mouth. Consequently, the indices were not measured at identical sites, which may contribute to differences in absolute values. In quadrants assessed with both indices, BOMP was recorded prior to BOPP. Importantly, the more invasive probing used for BOPP was intentionally performed second to minimize any increase in bleeding that could result from repeated probing. Each index was evaluated by a different examiner, and bleeding scores are sensitive to probing force and technique; although inter‐ and intra‐examiner calibration data were not collected, BOPP measurements were obtained using a force‐controlled probe to standardize pressure, whereas BOMP does not require a force‐controlled device, as previously demonstrated.^29^ Despite these considerations, the relative distribution and trends captured by both indices are consistent and provide meaningful insight into gingival bleeding patterns in this cohort.

### 4.5 Plaque

The regression models provided further insights into the factors influencing clinical parameters of gingival bleeding. PI was positively associated with both BOMP and BOPP (Tables 3 and 4), indicating that a higher level of plaque accumulation is linked to increased gingival inflammation. This finding is consistent with the well‐established role of dental plaque in the pathogenesis of gingivitis.^8^, ^30^, ^50^

### 4.6 Age

Age was negatively associated with both BOMP and BOPP, suggesting that younger individuals in this cohort exhibited higher levels of gingival inflammation (Tables 3 and 4). This contrasts with other research, such as Matsson and Goldberg,^51^ who found the opposite result when comparing children with young adults. A possible explanation for the negative association between age and bleeding tendency in our study could be behavioral factors such as oral hygiene practices or hormonal influences in younger adults.^52^

### 4.7 Sex

Interestingly, while sex showed a positive association with BOPP, it was not statistically significant (Tables 3 and 4). This indicates that although males tended to have higher BOPP scores, the difference was not substantial enough to be conclusive. Further research with a larger sample size may be needed to clarify the role of sex in gingivitis severity.

### 4.8 Vitamin C dose

In the Netherlands, a daily intake of at least 75 mg of vitamin C is recommended.^53^, ^54^ In this study 190 out of 262 participants (73%) met or exceeded this threshold (Table S1) and none had an intake below 10 mg/day, a level commonly used to indicate severe deficiency. Consequently, the present study primarily assesses variability within a largely vitamin C‐replete population. If the modulatory effects of vitamin C on gingival inflammation are most pronounced under conditions of deficiency or suboptimal status, our analysis may be insufficiently sensitive to detect such relationships. This consideration may, at least in part, account for the absence of a significant association between reported vitamin C intake and gingival bleeding in this cohort. While the average intake among participants was sufficient to prevent something as severe as scurvy, it may not have been high enough to exert a protective effect against increased gingival bleeding.^55^ Previous studies have similarly failed to demonstrate a clear association between vitamin C intake and gingival bleeding,^10^, ^11^, ^12^ although other studies found a relationship.^56^, ^57^ As long as bacterial plaque is present, gingivitis may persist regardless of vitamin C status, underscoring the importance of plaque control as the primary preventive strategy, as emphasized by Ismail et al.^58^

Notably, a 1946 study involving 120 men under the age of 30 found that supplementation with additional vitamins, including vitamin C, over a 5‐month period had no measurable clinical effect on gingival health. However, when gingivitis was first resolved through local treatment, subsequent supplementation with 75 mg of vitamin C appeared to delay the recurrence of inflammation.^59^

### 4.9 Limitations and considerations

#### 4.9.1 Population

This study used a convenience sample of 262 young, systemically healthy non‐dental students with a mean age of 22.6 years and with a range from 18 to 32 years. All participants were systemically and orally healthy and were non‐smokers. These strict selection criteria may limit the observable impact of vitamin C intake, as effects are less likely to emerge in individuals without deficiencies or pre‐existing oral health problems. In other words, since participants already have a good to reasonably good gingival health, it appears in hindsight that to detect any associations of vitamin C intake on gingival bleeding which was used as a surrogate marker for periodontal health is inherently challenging.

Although the participants are described as orally healthy, inclusion was based solely on a DPSI score minus ≤ 3. This threshold allows probing depths of up to 5 mm in the absence of recession and does not constitute a comprehensive periodontal diagnosis. Consequently, the cohort may have included individuals spanning the spectrum from periodontal health to localized or generalized gingivitis, and potentially even early‐stage periodontitis. However, given the young age and generally healthy systemic profile of the study population, the likelihood of including participants with established periodontitis is low.^60^ Because a full periodontal assessment, including clinical attachment level measurements, was not performed, the precise periodontal status of the participants cannot be definitively ascertained. This potential heterogeneity represents a limitation, as variation in underlying periodontal conditions may have influenced bleeding outcomes.

#### 4.9.2 FFQ and vitamin C intake

Another limitation of this study is the use of FFQs to assess dietary vitamin C intake. FFQs are among the primary tools used for collecting dietary data, especially in epidemiological research. These questionnaires are designed to assess long‐term dietary habits and are relatively cost‐effective to administer. However, this method is subject to recall bias and may not accurately reflect actual nutrient consumption. FFQs rely on a predefined list of foods and depend on individuals' ability to accurately recall their dietary intake over extended periods. These constraints can introduce errors in estimating usual dietary intake.^61^ Although the FFQ has been validated against circulating biomarkers, demonstrating a reasonable concordance between reported intake and measured nutrient concentrations, dietary intake remains an indirect proxy for physiological exposure. Inter‐individual variability in nutrient absorption, metabolism, and supplement bioavailability may result in discrepancies between the quantities reported in the questionnaire and the actual systemic availability of the nutrient.

Furthermore, although the FFQ included questions on supplement use, the available data did not allow differentiation between vitamin C obtained from foods and from supplements, nor quantification of the proportion of participants using vitamin C supplements. Consequently, potential differences between supplement users and non‐users could not be explored.

It should be noted that the FFQ captures dietary intake over the preceding 4 weeks, which may be insufficient to fully reflect longer‐term nutritional influences on gingival tissue remodeling. While this timeframe aligns with the biochemical turnover of collagen, the formation of mature, functionally organized collagen bundles may require a longer period to manifest in tissue‐level outcomes. Nonetheless, a 4‐week assessment is generally considered a reasonable indicator of habitual intake in epidemiological studies, providing a valid, if somewhat short‐term, estimate of typical vitamin C consumption.^21^, ^62^

### 4.10 Sensitivity analysis and detectable effect size

With *n* = 262, the study had 80% power (*α* = 0.05) to detect associations of approximately *r* ≥ 0.17 (partial *R* ^2^ ≈ 0.03) for vitamin C in both bleeding outcomes. The observed effects were substantially smaller, indicating that any true association is likely to be very small.

### 4.11 Further research suggestion

Overall, in the current observational study we could not find an association of vitamin C intake with gingival bleeding within a young, healthy college student population. Future studies should consider longitudinal designs to assess the impact of sustained vitamin C intake and other dietary factors on gingival health over time. Additionally, exploring the interplay between oral hygiene practices, dietary habits, other lifestyle factors (e.g., “stress”) and genetic predispositions could provide a more comprehensive understanding in the variation of existing gingivitis among students.

## 5 CONCLUSION

In this non‑deficient young adult study population, no significant correlations were observed between vitamin C intake and gingival bleeding tendency, measured with the clinical parameters BOMP and BOPP. Regression analysis indicated a positive association between PI and gingival bleeding on probing, and a negative association between age and gingival bleeding on probing.

## AUTHOR CONTRIBUTIONS

**Thijs M. H. de Jong**: Conceptualization; formal analysis; methodology; visualization; writing—original draft; writing—review & editing. **Dagmar E. Slot**: Conceptualization; funding acquisition; investigation; writing—original draft; writing—review & editing. **Cees Valkenburg**: Formal analysis; methodology; writing—review & editing. **Bruno G. Loos**: Conceptualization; funding acquisition; writing—original draft; writing—review & editing. **Fridus A. van der Weijden**: Conceptualization; formal analysis; funding acquisition; methodology; writing—original draft; writing—review & editing. All authors gave final approval and agreed to be accountable for all aspects of the work ensuring integrity and accuracy.

## CONFLICT OF INTEREST STATEMENT

The authors declare no conflicts of interest.

## ETHICS STATEMENT

The medical ethics committee of the Academic Medical Centre of Amsterdam approved the protocol (2012_210#B2012406), and it is registered in the Dutch Trial Register (NTR3649). Informed consent forms were signed by all participants.

## DECLARATION OF GENERATIVE AI AND AI‐ASSISTED TECHNOLOGIES IN THE WRITING PROCESS

During the preparation of this work the authors used Microsoft Copilot (based on the GPT‐4 architecture, Microsoft Corporation, Redmond, WA, USA) in order to improve the grammar and to rewrite sentences. After using this tool, the authors reviewed and edited the content as needed and take full responsibility for the content of the publication.

## Supporting information



Supporting Information

## Data Availability

The data used to support the findings of this study are available from the corresponding author upon request.
