# Supplementary material for: Exploring the association between vitamin C intake and gingival bleeding tendency in healthy, non‑deficient young adults
Source: J Periodontol. 2026 Jun 4;97(9):1910–23. doi: 10.1002/jper.70142 (PMC13569008; doi:10.1002/jper.70142)
Supplement: Supplementary file 1 [file JPER-97-1910-s001.docx]

**TABLE S1** | Vitamin C intake categories by sex.

| Sex | Vitamin C intake | Subjects (n) | Subjects (%) |
| --- | --- | --- | --- |
| Female | Very low (< 10 mg/day) | 0 | 0 |
|  | Low (10–74 mg/day) | 40 | 33.9 |
|  | Adequate (≥75 mg/day) | 78 | 66.1 |
| Male | Very low (< 10 mg/day) | 0 | 0 |
|  | Low (10–74 mg/day) | 32 | 22.2 |
|  | Adequate (≥75 mg/day) | 112 | 77.8 |

**TABLE S2** | Categorization gingival bleeding scores for BOMP and BOPP.

| Gingival bleeding index | Score | Subjects (n) | Subjects (%) |
| --- | --- | --- | --- |
| BOMP* | <0.1 (healthy) | 30 | 11.5 |
|  | 0.1-0.3 (localized) | 79 | 30.2 |
|  | ≥0.3 (generalized) | 153 | 58.4 |
| BOPP | <0.1 (healthy) | 0 | 0.0 |
|  | 0.1-0.3 (localized) | 23 | 8.8 |
|  | ≥0.3 (generalized) | 239 | 91.2 |

* Because BOMP scores range from 0 to 2 (0 = no bleeding, 1 = pin‑prick bleeding, 2 = excessive bleeding) within 30 seconds after probing, a mean score of 0.1 corresponds to approximately 5–10% bleeding sites, and a score of 0.3 corresponds to roughly 15–30% bleeding sites.
